# Electricity in Its Relations to General Practice

**Published:** 1882-07-20

**Authors:** Thomas F. Houston

**Affiliations:** Clarkesville, Ga.


### Southern Medical Record:

EDITORS:

T. S. POWELL, M.D. W. T. GOLDSMITH, M.D. R. C. WORD, M.D.

*R. C. WORD, M.D., Managing Editor.*

☞All Communications and Letters on Business connected with the RECORD must be addressed to the Managing Editor.

VOL. XII. ATLANTA, GA., JULY 20, 1882. No. 7.

### ORIGINAL AND SELECTED ARTICLES.

### ELECTRICITY IN ITS RELATIONS TO GENERAL PRACTICE.

BY THOMAS F. HOUSTON, M. D., CLARKESVILLE, GA.

INTRODUCTION.

There is no agent in the Materia Medica of greater importance, or that can be successfully applied to more varied diseases, than this; and there is not one of which the bulk of medical men show more ignorance. There are several reasons for this fact: Our colleges almost ignore the subject, and after entering the busy round of professional life, its importance not being understood, they neglect it for studies that they consider of more practical importance. Speaking of his specialty, Emmet says: "Success in the treatment of diseases of women lies wholly in attention to minute details." This also is the secret of successful electro-therapy. The choice, direction and strength of the current, the duration of the sitting, the size and location of the electrodes, etc., are all elements of success or failure. Yet, how is the busy practitioner to acquire this practical knowledge, without which it is labor thrown away—success being the veriest accident—and not only casts unmerited shame upon this valuable remedy, but degrades the physician to the level of the quack. Take up that most magnificent treatise, Beard and Rockwell on Electricity, and it will take months of arduous study to acquire a practical knowledge of its therapeutical application.

The chief objection to the various epitomies, and I have five of the best on my table, is that they fail in being elementary enough. Electricity is so profound a science, and has so many technicalities, that it can almost be said to have a language of its own. The scientists who write these manuals are so familiar with their use and meaning that they forget to come down to the level of the uninitiated.

My papers upon this subject will not present treasures of original research and profound thought, nor are they intended for the perusal of electro-specialists, but as stepping-stones, as an alphabet, to the larger and more complete works. As I am limited in space, I cannot discuss conflicting theories and opinions, but must confine myself to those points that have a practical bearing upon general medicine. And if for the same reason—want of space—I should seem dogmatic and egotistical, this will serve as my explanation and apology. With this introduction, let us plunge *in medias res* and take up

ELECTRO-PHYSICS.

Electricity, like heat and light, is a variety of molecular motion. For the sake of illustration, we compare it to a fluid—hence we say the current flows. This motion may be *constant*—the galvanic current; or interrupted—the faradic; or a succession of sparks—the frictional. We will take them up in the order mentioned, reserving the discussion of frictional electricity to a separate chapter.

THE GALVANIC OR CONSTANT CURRENT.

The simplest means of producing the galvanic current is to plunge two dissimilar metals, connected by a wire--say copper and zinc—into a dilute acid, sulphuric for instance. This is called a galvanic *couplet*, or *element*, or *combination*, or *cell*. The impurities in commercial zinc prevent the formation of a current, hence it is necessary to amalgamate it, *i.e.*, rub it with quicksilver after washing it in dilute sulphuric acid. Two plates of the same metal will not produce a current, because the chemical action taking place at both poles of the battery, the current that commences to be generated by each is neutralized by that of the other. One of the plates in the *element* is the *generating plate* or *pole;* the other the *conducting plate* or *pole*. In the example given above the zinc is the *generating* plate; sulphate of zinc being found is dissolved in the acid, this chemical action evolves a current of electricity which passes through the fluid to the copper (the conducting plate) and around the wire to the zinc; thus completing the circuit. For convenience of discussion we call the conducting plate and its wire the positive pole, and the generating plate and its wire the negative pole. Hence we say the current (meaning that portion external to the battery) flows *from the positive to the negative pole.* This fact is of great clinical importance, and I will discuss it fully when speaking of *electro-physiology.* The *volume of the current* is in proportion to the amount of chemical action, and this is determined by the amount of surface area in the generating plate. The *intensity of the current* has reference to its capability of overcoming resistance. All substances offer a certain amount of resistance to the passage of the electric current, and this is what is meant when we say a body is a good or bad conductor. It has been determined by actual experiment that the resistance offered to the passage of the current from one hand to the other is seven times greater than that of the trans-Atlantic cable (vide Beard & Rockwell, page 82) or four million times that of a copper wire. To illustrate the difference between volume and intensity: Take twenty elements like that described above, connect the positive pole of the first with the negative pole of the second, and so on completing the circuit by the union of the first and twentieth cells. As the current traverses the cells it is not increased in volume, but each cell adds its pro rata to the intensity of the passing current. So we get a current of small volume but great intensity, therefore capable of producing valuable therapeutic effects. This can be reversed: connect the similar pole of each cell to an electrode, and we get a current of great volume but of so low a tension that it is therapeutically worthless. The simplest form of battery is the one already described, having two metals and an acid solution. It is also the most portable, hence most useful to the general practitioner. For this reason we will confine our attention to the single fluid elements. The Grenet cell is the best of these. Zinc and carbon are the elements, and an acid solution of bichromate of potash the fluid. I will describe this fully in the future, and will leave the galvanic and briefly discuss

### The Faradic Current.

The battery which produces this current consists of a single galvanic element, generally a Grenet cell. It consists of a glass cup containing an acid solution of bichromate potash. Fastened to the lid are two plates of carbon that are always immersed in the fluid. Between them is a plate of amalgamated zinc attached to a hinged rod of brass. When the battery is to be used the zinc is lowered into the fluid, chemical action commences and the current flows to the carbon and completes the circuit. The connecting wire is insulated, and is sufficiently long to form a helix—called *the induction coil.* In the center of the induction coil is another coil of soft wire wound around a bundle of insulated soft iron rods of the size and shape of knitting needles. The inner coil is called the *secondary coil,* and the bundle of insulated rods *the core.* At the instant the circuit is completed in the *induction coil* there is a *momentary* but *entirely independent current* generated in the *secondary coil* running in the *opposite* direction. At the instant that the primary current is interrupted, there is again a current generated in the secondary wire, this time running in *the same direction* as the primary. Let us now introduce into the primary current a rheotome, or current interrupted, and we get in the secondary wire a to and fro current that is far stronger than that of the single galvanic cell and is of great therapeutic value. All secondary currents possess the power of generating in a third coil a tertiary current flowing in an opposite direction and of lower tension and less volume. This can be repeated again and again, the direction of each current being reversed and decreased in strength until exhausted. When the connecting wire of a galvanic element is insulated and wound around a bar of soft iron while the current is passing, it is converted into a maget, and it is possible to make them of very great power for the length of time that the current is passing—its power merely depending upon the volume and intensity of the electric current. This is called an *electro-magnet.* At the instant that the encircling current is closed and opened an electro-magnet possesses the power of generating, by induction, a *momentary* current of electricity in any coil of wire that is near enough to be affected by it.

The rheotome, or current interrupter, is generally some modification of Neef's hammer. This hammer terminates in a spring whose pressure holds it in place, thus completing the circuit. At the instant that the circuit is completed *the core* becomes a magnet and attracts the hammer, this breaks the circuit and stops the first induced current, and the second is generated. As the circuit is broken the core becomes demagnetized and the spring throws the hammer back to its place, again completing the circuit. Again the first faradic current is generated, the core becomes a magnet, acts on the hammer, etc. The first induced current is the weaker of the two from this fact. The core becoming a temporary magnet by the closure of the circuit, generates in the secondary coil a current flowing in the same direction as the primary current, *i.e.*, opposite to the induced but occupying the same wire. The opposite direction of these two currents neutralize to a certain extent each other. Thus the first induced current is made weaker. On the other hand, the second induced current is strengthened, because upon the opening of the circuit the core again generates in the secondary coil a current flowing in the same direcion as before and parallel to the second induced current. Thus we have in the secondary coil, upon the closure of the circuit, two currents generated flowing in opposite directions impeding each other; in the opening of the circuit two currents flowing in the same direction strengthening each other. At the same time that these currents are being generated by the core the turns of the primary coil act upon each other by induction, and other currents are produced, called by Faraday the "extra currents." These are collected in the faradic batteries now in use, and are what is meant when we speak of the "primaay current of Faradism."

With this exposition of electro-physics, I will leave the subject, simply adding this thought: Electro-physics is the foundation-stone of successful electro-therapy, and unless we lay our foundation broad and deep, and make the next row of stones equally as secure in *mastering* electro-physiology, our edifice will always be shaky, and (to drop the metaphor) we can hope for but little success.

